# The Current Status of Analytical Methods Applied to the Determination of Polar Pesticides in Food of Animal Origin: A Brief Review

**DOI:** 10.3390/foods11101527

**Published:** 2022-05-23

**Authors:** Emanuela Verdini, Ivan Pecorelli

**Affiliations:** Pesticides and Mycotoxins Laboratory, Chemistry Department, Istituto Zooprofilattico Sperimentale dell′Umbria e delle Marche “Togo Rosati”, 06126 Perugia, Italy; i.pecorelli@izsum.it

**Keywords:** polar pesticides, glyphosate, food of animal origin, chromatographic techniques, non-chromatographic techniques

## Abstract

The use of high polar pesticides such as glyphosate and metabolites has increased due to their low cost, low persistence in the environment and high effectiveness. The use of glyphosate is currently permitted in the European Union until 15 December 2022. However, the possible toxic effects on human health and the environment are under debate. Their widespread application on various crops might lead to residues in food intended for animal consumption. For this reason, the Commission, implementing Regulation (EU) 2021/601, recommends the analyses of polar pesticides, not only in matrices of plant origin, but also in those of animal origin such as fat, liver, milk and eggs throughout the years 2022, 2023 and 2024. The determination of polar pesticides is hampered by their chemical nature, which poses challenges both in the instrumental detection (poor column retention, low molecular weight MS/MS fragments, etc.) and in the management of matrix effects, which may vary significantly from matrix to matrix within the same food commodity group. For these reasons, nowadays, there is a limited number of methods for the detection of polar pesticides in food of animal origin. This brief review discusses the different approaches for the simultaneous determination of polar pesticides in food of animal origin using both chromatographic and non-chromatographic techniques.

## 1. Introduction

Polar pesticides are widely used in agriculture due to their low cost and high effectiveness, as they increase crop yields and allow the growth of fruits with desirable characteristics for the consumer [1,2].

**Figure 1:**
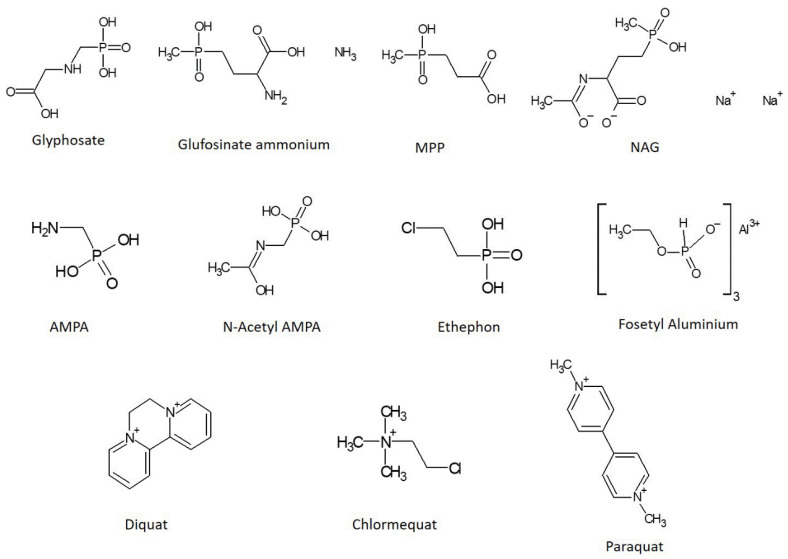
Chemical structures of glyphosate, glufosinate ammonium, [3[hydroxy(methyl)phosphinoyl]propionic acid (MPP), N-acetyl-glufosinate (NAG), aminomethylphosphonic acid (AMPA), N-acetyl-AMPA, fosetyl aluminum, ethephon, diquat, chlormequat and paraquat.

Glyphosate is the best-known pesticide belonging to this class. In addition to glyphosate, there are other highly polar pesticides, such as glufosinate, fosetyl, ethephon and their metabolites, which constitute an extremely challenging group of molecules to be analyzed due to their physicochemical properties [3]. The chemical structures of some polar pesticides are shown in Figure 1.

While the use of glyphosate in agriculture is currently approved in the EU, many issues relating to its toxicity and carcinogenicity have emerged over the years. As the study by Ghandi et al. [4] reports, it has been observed that glyphosate is negatively affecting the ecosystem due to the exposure of non-target species, increasing the number of pathogenic microorganisms or causing diseases in both humans and animals such as renal and respiratory dysfunction, metabolic acidosis, tachycardia in humans, malformations in newborns, hormonal imbalances and oxidative stress for animals. On the other hand, in the various studies reported in this work, an important aspect that should be taken into account is related to the toxicity of the adjuvants used for the technical formulations of glyphosate, as well as the transformation products of glyphosate such as aminomethylphosphonic acid (AMPA), which makes the evaluation of toxic effects even more complex. In 2015, the International Agency for Research on Cancer (IARC) reported that glyphosate is a probable carcinogenic for humans [5]. In 2017, however, a scientific opinion of the Committee for Risk Assessment (RAC) of the European Chemicals Agency (ECHA) concluded that glyphosate, while causing serious eye damage and being toxic to aquatic life with long-lasting effects, has not been proven to be carcinogenic, mutagenic or to have adverse reproductive effects because the available scientific evidence did not meet the criteria to classify it as a probable carcinogen [6]. Likewise, the European Food Safety Authority (EFSA) concluded that AMPA presents a similar toxicological profile to glyphosate. No toxicological data were available on N-acetyl-glyphosate and N-acetyl-AMPA. The need for information on this issue was identified as a data gap [7]. In 2019, the Glyphosate Renewal Group (GRG) formally applied to renew the approval of glyphosate for use after the current approval that will expire at the end of 2022 [8].

Due to the fact that the vast majority of forage or grains treated with polar pesticides are consumed by farm animals [9], the presence of these molecules is of great concern for human health as a result of the possible transfer of pesticides, their metabolites or by-products in animal tissues. 

To monitor the presence of polar pesticides in food and ensure compliance with the EU maximum residue levels (MRLs) [10], on 13 April 2021, the European Commission promulgated a Regulation that provides a multi-year control plan of pesticide residues in matrices of animal and plant origin. In the context of polar pesticides, the analysis of glyphosate and glufosinate ammonium in bovine, pig and poultry fat, chicken eggs, bovine liver and cow milk was planned over the years 2022, 2023 and 2024 [11].

When the necessary MRLs are set, including metabolites in the complex residue definition. For products of animal origin, the lack of information regarding the presence of glyphosate and its metabolites in these matrices means that MRLs are still provisional [12].

Polar pesticides are mainly analyzed with so-called single residue methods instead of multi-residue methods such as QuEChERS (quick, easy, cheap, effective, rugged and safe), commonly used for the analysis of pesticides in foods of animal and plant origin due to the high degree of polarity of these molecules, which poses challenges either during the extraction/purification phases or in the instrumental detection.

In fact, these molecules require the use of long and complex cleaning procedures that sometimes involve anionic and cationic exchange columns [13] to mitigate the effect of the matrices that may contain many interferences [14]. In particular, in matrices of animal origin, lipids are generally co-extracted along with the analytes of interest [15]. Moreover, the absence of chromophore or fluorophore groups in the molecules requires the use of derivatization techniques for the determination of these residues by liquid chromatography coupled with fluorescence or photometric detectors. Specific chromatographic columns must be used for these analyses (anion exchange, hydrophilic interaction liquid chromatography (HILIC), porous carbon graphite (PGC) and mixed-mode columns) since typical reversed-phase C18 or C8 columns do not have a satisfactory retentive efficiency due to the charges that polar pesticides possess in the aqueous phase [16].

The aim of this review is to provide an overview of the analytical methodologies available in the analytical determination of polar pesticides in food of animal origin.

Most of the works in the literature concern the analyses of polar pesticides in food of plant origin and environmental samples focusing exclusively on glyphosate [17]. Although Avino et al. [18] published a review in 2020 on polar pesticides in both vegetables and food of animal origin, the present work addresses the problem of analyzing these analytes in samples of animal origin in a broader and more widespread way. In particular, it provides more specific insights into each chromatographic and non-chromatographic technique, extraction procedure and validation data; in conclusion, a critical evaluation of the overall performance of each method.

## 2. Analytical Methodologies

### 2.1. Determination of Polar Pesticides by Enzyme-Linked Immunoabsorbent Assay (ELISA)

Krüger et al. [19] used an ELISA kit (Abraxis, USA) for the detection of glyphosate in samples from German dairy cows, in particular liver [*n* = 41], kidney [*n* = 26], lung [*n* = 23] and muscles [*n* = 6]. For the preparation of the extracts to be analyzed, the samples were triturated, diluted by adding water according to the retention rate of the matrices, heated at 100 °C for 10 min, homogenized and frozen at −80 °C for 8 h. They were then thawed at 40 °C and centrifuged at 10,000× *g* for 10 min. The supernatant was filtered with an ultra-centrifugal filter with a cut-off of 3000 Da and again centrifuged (10,000× *g*) at 20 °C for 10 min. The samples were tested according to the manufacturer′s protocol. Test validation of ELISA was conducted in comparison with GC-MS techniques, and a value of 0.96 for Spearman rank-order correlation was obtained. For the recovery study, 8 meat samples were spiked with 100 μg of glyphosate. Minimum and maximum concentrations obtained were 75.25 and 164.56 (µg/g), respectively. The mean was 109.26 and standard deviation was 30.13 (µg/g). The recovery mean was 91%. 

In the study of John et al. [20], the same ELISA kit was used for the determination of glyphosate in several foods, including milk, beef and fish. The standard curves were constructed using a series of standard samples provided by the kit (0.075–4.0 ppb). In three measurements, the values of the correlation coefficient R2 were 0.92, 0.96 and 0.99. The samples were centrifuged twice or sliced, homogenized in laboratory water and centrifuged for milk, beef and fish, respectively. Then, the supernatant was collected. In this study, the results were difficult to interpret; the data were not consistent with the results reported by others, and there was a lack of validation data, as also highlighted in the article by Vicini JL et al. [21].

### 2.2. Determination of Polar Pesticides by GC Coupled to Mass Spectrometry (MS/MS)

The technique for GC quantification requires derivatization with compounds able to convert glyphosate and AMPA in a single reaction step only to derivatives that are sufficiently volatile for GC/MS analysis [22].

In the work of Alferness et al. [23], samples of beef, muscle, kidney, cow milk and chicken eggs were analyzed for glyphosate and AMPA determination. The samples were extracted with HCl and chloroform, or with acetic acid in the case of milk, and purified using Bio-Rad Poly-Prep disposable columns containing 50WX8 AG resin (H+ form) (cation exchange clean-up). The derivatization was based on trifluoroacetic anhydride (TFAA) and 2,2,3,3,4,4,4-heptafluoro-1-butanol (HFB). HFB was selected to obtain derivatives with high mass fragments that could be selectively detected, with a minimum of interfering peaks.

The derivatization process was found to be highly reproducible. For this study, eight water samples were fortified with 0.2 pg/L; then, a GC injection was made from each of the eight derivatized samples. Coefficient of variation (CV) values of 4.8 and 6.4% were obtained for the glyphosate and AMPA derivatized samples, respectively. The same study was also carried out for samples of animal origin, obtaining a precision similar to that of water.

The analytical system consisted of a Hewlett-Packard (HP) 5890 gas chromatograph equipped with an HP 5970A MSD, HP 7673 automatic sampler injector, with a split-splitless inlet system operated in the splitless mode connected to a Finnigan MAT Model TSQ 70 mass spectrometer. Expected structures of the derivatives were confirmed by electron impact (EI) mass spectra of the compounds using the mass selective detector in the scan mode. This study underlines the fact that to improve instrumental performance, it was necessary to set an oven with a temperature program that foresees a lower initial temperature and an intermediate temperature ramp. In addition, the type of inlet liner used was also important. Double-restrictor liners were preferred. For the validation study, 3 samples for each matrix were spiked at LOQ (limit of quantification) and 3 at LOQ ×10 levels. The range was 0.01–1.0 mg/kg for each analyte. The mean recovery was 98% and 89%, with a coefficient of variation (CV%) equal to 8 and 11% for glyphosate and AMPA, respectively. Moreover, an interlaboratory study was conducted with two external groups, and this confirmed the goodness of the method.

Krüger et al. [18] did not report validation data and only Spearman rank order coefficients between ELISA and GC-MS.

Samples were prepared in the same way as those for ELISA analysis, and they were measured according to the procedure of Alferness and coworkers with some modifications. An internal standard solution containing ^13^C_2_-^15^N-glyphosate was added to 100 µL of each sample extract to which 1 mL of acetonitrile (ACN) had previously been added. Subsequently, the samples were dried down using a vacuum centrifuge. For derivatization, 0.5 mL of 2,2,2-trifluoroethanol and 1 mL of freezing cold (−40 °C) TFAA were cautiously added to the residue. After sonication and heating, the resulting mixture was dried down, obtaining an oleous residue, which was then redissolved with 200 µL of CAN.

The samples were measured using a gas chromatograph 7890 A equipped with a split/splitless injector connected to a 7000 Triple-Quad mass spectrometer operating in the chemical ionization (NCI)-mode (both instruments from Agilent Technologies, Waldbronn, Germany).

### 2.3. Determination of Polar Pesticides by Flow Injection Combined with Tandem Mass Spectrometry (MS/MS) (FI-MS/MS)

Mol et al. [24] sought a quick, easy and efficient technique for the determination of highly polar SRM pesticides, eliminating LC separation and providing a solution for MS/MS capacity constraints. Several foods, such as milk, kidney and many analytes, namely ethephon, fosetyl-Al, glufosinate, glyphosate, aminomethylphosphonic acid (AMPA), maleic hydrazide, chlormequat, diquat, mepiquat and paraquat, were included in this study.

For the extraction procedure, 10 mL of milliQ water was used by shaking end to end for 30 min. For paraquat and diquat, 1% formic acid was used. The extract was then centrifuged, diluted at least 10 times to obtain a matrix load equal to 0.05 g/mL and finally filtered using a mini-uniprep PTFE. A pump system from Shimadzu (DGU-20A3 degasser; SIL20 AC XR autosampler; LC-20 AD XR pump) (Shimadzu,‘s-Hertogenbosch, The Netherlands) and an AB Sciex QTRAP 5500 mass spectrometer equipped with an electrospray (ESI) source (AB Sciex, Zuidplas, The Netherlands) were used. The injector was directly connected to the source.

The method was optimized by studying various injection solvent compositions and various purification techniques to inhibit the matrix effect. MeOH acidified at 0.1% was found to be the best solvent for chlormequat, paraquat, mepiquat and diquat, while MeOH 90% + 0.1% NH_3_ for the others. Liquid-liquid separation with dichloromethane or hexane, dispersive solid-phase extraction (SPE) with C18 or graphitized carbon black (GCB) and ion exchange SPE cartridges were studied for purification, but no significant improvement was achieved. A 10 times dilution of the initial extract only was adopted. Moreover, using this technique, the configuration (capillary dimensions) and conditions (injection volume and flow rate) used were also very important. In this study, a 1 m (the shortest possible length to connect the autosampler with the source without changing the setup of the LC–MS/MS equipment) capillary with 0.13 mm internal diameter, a 1 μL injection volume and a flow rate of 400 μL/min were used.

The validation study was conducted for apple, lettuce and wheat matrices only. Quantification was made using both matrix match calibration and isotopically labeled internal standards, where the latter showed a better precision and compensation of the matrix effect. The method detection limits were 0.02 mg/kg for chlormequat and mepiquat and 2 mg/kg for maleic hydrazide and were comprised in the 0.05–0.2 mg/kg range for most of the other pesticide/matrix combinations. The applicability of FI–ESI–MS/MS for detection of various SRM-pesticides in animal origin matrices was demonstrated by the chromatogram in the paper that showed pesticide/matrix combinations for blank and spiked samples but only for cow milk spiked with chlormequat at 0.05 mg/kg and swine kidney spiked with glyphosate at 0.2 mg/kg and 1.0 mg/kg.

### 2.4. Determination of Polar Pesticides by Ion-Chromatography High-Resolution Mass Spectrometry IC-HRMS

In the study of Chiesa et al. [16], an analytical method for the determination of glyphosate, glufosinate and AMPA in fish (bass) and bovine muscle was developed and deeply validated according to the SANTE guideline [25]. All samples were spiked with the internal standard (ILIS) of glyphosate. A very simple extraction procedure was applied with no purification steps for all matrices. In particular, 3 mL of methanol (MeOH) and 7 mL of acidified deionized water (1% formic acid) were added to one gram of each sample, after which they were mixed and sonicated. Finally, after centrifugation, 1 mL of the supernatant was filtered with a mixed cellulose syringe filter (0.45 μm) and injected into the IC-MS/MS. The analyses were performed by an ionic chromatography (IC) Dionex ICS-5000+ system (Sunnyvale, CA, USA) made up of a dual pump (DP), a conductivity detector (EG), a detector/chromatography module (DC) and an autosampler (AS-AP) coupled with a Thermo Q-Exactive Orbitrap™ (Thermo Scientific, San Jose, CA, USA), equipped with heated electrospray ionization (HESI) source (negative mode). The column was a Thermo Scientific Dionex IonPac AS19-4 μm (2 × 250 mm, 4 μm) with a guard column Dionex IonPac AG19–4 μm (2 × 50 mm, 4 μm). The spectra acquisition was obtained through a full scan experiment (FS) (scan range *m*/*z* 50–250, combined with data independent acquisition mode (DIA). The resolving power was set at 70,000 and 35,000 full width at half maximum (FWHM) for FS and DIA experiments, respectively. Instrument calibration was conducted in every analytical session. From the validation study, it is clear that these means are very efficient for the three matrices. The LOQs were 5.38 and 6.44, 5.08 in fish and 6.47, 4.36 and 6.25 µg/kg for AMPA, glyphosate and glufosinate in bovine muscle, respectively. All calibration curves showed good linearity (R2 > 0.99), and the recoveries obtained were 95 and 99% for AMPA, 93 and 107% for glyphosate and 96 and 106% for glufosinate; all CV% were comprised between 4.2 and 13.12%.

### 2.5. Determination of Polar Pesticides by High-Performance Liquid Chromatography (HPLC) Coupled with Tandem Mass Spectrometry (MS/MS)

#### 2.5.1. Determination of Derivatizated Samples

The most common derivatizing agent used was fluorenylmethylchloroformate (FMOC-Cl).

The acquity UPLC BEH C18 column was used in two works [26,27] for the determination of glyphosate and AMPA in cow milk in the former and of glyphosate in chicken, pig and hen liver in the latter. In both cases, internal standards were used for quantification and FMOC-Cl as the derivatizing agent.

In the work by Ehiling et al. [26], due to the presence of the free phosphate group in the derivatized analytes, peak tailing was observed. To avoid this problem, the column was conditioned with 0.1% phosphoric acid for 30 min before use. The addition of HCl at pH 1 was used to overcome the problem of the complexation of glyphosate with different cations, and the samples were purified by the addition of methanol only. For further details, the extract to be derivatized was prepared by adding 50 μL of hydrochloric acid (34–37% wt%) and 2 mL of methanol to 1 g of sample, then stirred and centrifuged. For derivatization, 0.6 mL of borate buffer was added to 0.3 mL of supernatant, followed by 0.5 mL of FMOC-Cl solution (1.5 mg/mL in ACN). The tubes were shaken and incubated for 30 min at room temperature. Finally, 60 μL of formic acid (98% wt%) were added, and the extracts were filtered through 0.2 μm PTFE syringe filters.

In Szternfeld′s work, [27] unlike the previous study, a sample purification procedure was applied. After extraction with methanol/water (20/80 *v*/*v*), 5 mL of dichloromethane and 20 mL of acetic acid (50%) were added to the supernatant, obtained after centrifugation, for the removal of fat and proteins. A second extraction was carried out with the further addition of dichloromethane to completely eliminate the fat. The extract obtained was finally purified by solid-phase extraction (SPE) with weak anion exchange cartridges to remove primary and secondary amine interferences extracted from the matrix. The eluate was dried down at 55 °C and then resuspended in 1 mL of Milli-Q water by ultrasound for 10 min at room temperature. For derivatization, 1 mL of borate buffer at pH 9 and 1 mL of FMOC-Cl were added to the extract and left in the dark for 45 min. After that, the derivatization was stopped by adding 2 mL of dichloromethane. The final extract was then filtered and injected into LC-MS/MS. The presented methods were linear with R2 > 0.99 in a concentration range from 5−500 ng/mL for the first and from 0.020–0.500 mg/kg for the second work. The validation study of Ehiling et al. was conducted only for soy protein isolate, while for other matrices analyzed, including bovine milk, four replicates were spiked at 0.005 µg/g, and the recovery range was 86–118% with RSDs of 10%. In Szternfeld′s work, all matrices have been validated. To evaluate the precision and accuracy, matrices were spiked at two concentration levels, namely at LOQ (0.025 mg kg^−1^) and 10*x*LOQ (0.250 mg kg^–1^) levels. Recoveries were 101.1%, 94.9% and 114.5% for veal, swine and chicken livers, respectively, with RSD% comprised between 7.3 and 13.1.

An analytical method was developed by Li Bo et al. [28] for the determination of glyphosate and AMPA in various matrices, including chicken and pork muscles, using 1,2-^13^C^15^N glyphosate as an internal standard. Two consecutive extraction steps were conducted for sample preparation. LC-grade (100 mL) water and 50 mL of dichloromethane were added to 10 g of sample for the first step, while 50 mL of fresh LC-grade water was added for the second one. Finally, about 4.5 mL of water phase was mixed with 0.5 mL of pH adjusting solution. Cation-exchange (CAX) SPE cartridge was used for purification. The eluted solution was concentrated to dryness at 45 °C and redissolved in 1.0 mL of 5% borate buffer. To derivatize the sample, the final pH was adjusted to 9, and 0.2 mL of FMOC-Cl (1.0 g/L in acetone) were added and left to react overnight at room temperature. The reaction solution was filtered through a 0.45 μm pore membrane filter for subsequent HPLC-MS/MS analysis. The analysis was performed in positive ionization mode with a Supelco Discovery C18 (5 μm, 150 mm × 2.1 mm i.d) column, obtaining average recovery values from 80.0% to 104%, with RSDs ranging from 6.7% to 18.2% for glyphosate and AMPA concentrations at 0.05, 0.10 and 0.50 mg/kg.

Another derivatizing procedure described in the literature [29] involves 5-(dimethylamino) naphthalene-1-sulfonyl chloride (dansyl chloride) for the determination of glyphosate AMPA and glufosinate in bovine liver, kidney and milk matrices. Simple extraction was applied; 2 mL of ultra-pure water and 3 mL saturated aqueous ethylenediaminetetraacetic acid disodium salt dihydrate solution (~10% concentration) were added to 1 g of sample. After centrifugation, 180 μL of filtrated aliquots were used for derivatization; 20 μL of saturated sodium carbonate solution and 240 μL of dansyl chloride solution (1.5% mass concentration in 40:60 acetone-acetonitrile) were added and stored at 40 °C for 30 min. The final solution was acidified with formic acid solution (1.5% in ultra-pure water). The mixture was filtered, and 40 μL aliquots were injected. The performance of the proposed analytical procedure was compared for all matrices, with that of the QuPPe procedure proposed by the European Union Reference laboratory for pesticides using single residue methods (EURL-SRM) (described in the next paragraph 2.5.2) [30]. The determination of dansyl chloride derivatized analytes and their respective IS was performed with HRMS Orbitrap and low-resolution tandem mass spectrometric detection (LRMS) using a Luna^®^ C18 column (Phenomenex, Torrance, CA, USA), while the determination of underivatized analytes using a Waters anionic polar pesticide column, 100 × 2.1 mm, 5 μm particle size with low-resolution mass spectrometric detection. In the liver and kidney, when an HRMS Orbitrap instrument was used, LOQs in the 10–25 μg/kg range were observed. Meanwhile, when using LC-MS/MS (LRMS triple quad) instrumentation, higher LOQs, ranging from 10 up to 250 μg/kg, for derivatized analytes and 10 μg/kg for underivatized analytes were observed, respectively. This phenomenon was related to the increased background signal. Calibration was performed by adding analytes and isotopically-labeled internal standards to the reagent blanks, obtaining R2 values > 0.98 for all analytes. The recoveries were in the range of 80–120%, and uncertainties ranged from 4 to 33% for all analytes. The chromatograms of derivatized samples showed interferences when the triple quadrupole mass spectrometer was used; different Orbitrap and underivatized chromatograms were free from interference. Despite the good results obtained for the methods tested, high-resolution mass spectrometry should be used to resolve interferences and improve signal-to-noise ratios and absolute peak intensities. The median measurement uncertainty and standard deviation of the calibration curve slope were lower in the case of the derivatization-based procedure due to the better electrospray stability and the lower ion suppression compared to the results obtained with the Waters column.

#### 2.5.2. Determination of Underivatizated Samples

A multi-residue method for polar pesticides extraction and analysis was developed by EURL-SRM. The quick polar pesticide (QuPPe) method [30] was designed by a common extraction/purification procedure followed by different combinations of analytical column separations and LC-MS/MS methods for the simultaneous analysis of groups of polar pesticides. The extraction was carried out with water and methanol acidified with formic acid. Ethylenediaminetetraacetic acid tetrasodium (EDTA) solution was used as a chelating agent for the metals present in the matrices. All extracts were cleaned-up with acetonitrile and dSPE with ODS sorbent. Different levels were validated in the range 0.005–0.2 mg/kg for different groups of analytes and matrices. Butter fat, bovine liver, bovine kidney and swine muscle were validated with the addition of EDTA to extracts, while chicken eggs were validated without this step. Whole cow milk was validated either way. Quantification in the great majority of cases was performed with the use of isotopically labeled internal standards. All validated concentrations met the requirements for recovery (in the range 70–120%) and precision (RSD ≤ 20%) stated by the SANTE document [25].

A method for the analysis of diquat, paraquat and chlormequat (QUATs) in livestock animal products using UPLC-MS/MS was developed by Il Kyu Cho et al. [31]. The extraction procedure was split into two steps; 15 mL of 0.5% formic acid in acetonitrile and 5 mL of 0.5% formic acid in water were added to 5 g of sample. The first extract was centrifuged, and then the whole solution was transferred to another centrifuge tube. Ten milliliters of 0.5% formic acid in acetonitrile was added for the second extraction. The organic layers were eliminated by a gentle stream of nitrogen at 40 °C. The remaining aqueous solution was adjusted to 6.25 mL with 0.5% aqueous formic acid solution, and then an additional 6.25 mL of 0.5% formic acid solution in acetonitrile were added. Extract (2 mL) was loaded to an HLB LP cartridge, and the purified extract was combined with 0.4 mL of 0.5% formic acid solution in acetonitrile. Finally, 1 mL was transferred to a 2 mL plastic vial, followed by the addition of 10 μL of 5000 mM ammonium formate. The MS/MS determination was carried out in multi-reaction monitoring mode (MRM) with positive electron spray ionization (ESI+), and the chromatographic separation was carried out by hydrophilic interaction liquid chromatography column (HILIC). A matrix-matched calibration curve with a determination coefficient (R2) ≥ 0.991 was built to compensate for the matrix effect. The limit of detection (LOD) and LOQ of the method for the three compounds were 0.0015 and 0.005 mg/kg, respectively. Accuracy and precision of the method were calculated from the recovery and repeatability values and ranged from 62.4 to 119.7%, with a relative standard deviation of less than 18.8% for three levels of fortified standard (LOQ, 2 × LOQ and 10 × LOQ).

Jensen et al. and Zoller et al. [32,33] studied the development and validation of two independent extraction procedures for the quantification of glyphosate and AMPA in milk samples. Both methods used internal standard calibration and Bio-Rad Cation-H protection column for the chromatographic step. The first one consists of positioning samples in a 96-well plate, defatting by centrifugation and then purifying by methylene chloride, while the second one extracts analytes with H_2_O/MeOH, acidificates the solution and finally purifies samples with SPE HLB (60 mg). The results of the validation study of Jensen et al. showed recoveries within 88–114% with RSD < 7.4% for replicate values at fortification levels equal to 10, 25 and 2500 µg/L (ppb). The calibration curves present coefficients of determination (R2) > 0.999 for both transitions of glyphosate and AMPA in all matrices. All points on the calibration curve were within ± 20% of their respective nominal concentrations. The LOQ was set at 10 µg/L for both analytes. To evaluate performances, the method was checked in a second laboratory with different instrumentation. In this case, results were slightly different in terms of LOQ [25 mg/L (ppb)], mean recovery (between 88 and 99%) and precision (RSD values below 10.4%).

Zoller et al. employed only two replicates for their validation study. The LOQs were set at 0.5 µg/mL for glyphosate and at 1 µg/mL for AMPA, while the validation level was conducted at 4 µg/mL (greatly above LOQs). Average recovery values of 96 and 111% for glyphosate and AMPA were obtained, respectively, both with an RSD < 2%.

Narong et al. [34,35] published two papers regarding the determination of glyphosate and AMPA in milk and egg matrices, both suggesting 50 mM acetic acid/10 mM Na_2_EDTA for extraction and Oasis HLB cartridges (60 mg) for purification. Methylene chloride was also added to egg samples to extract the fat from egg yolk. Several mixed-phase mode chromatographic columns containing reverse-phase, anionic and cationic exchange properties were evaluated in the first study. In the end, the authors chose the Acclaim TrinityT Q1 (weak cation, weak anion/reverse phase) because this column has both cationic and anionic exchange properties and is therefore ideal for the analysis of both cationic and anionic pesticides. This column was also applied in the second study. The duration of the acquisition time in both methods was very short, 6 min for milk and 12 min for egg, respectively. For milk, experimental LOQs were 1, 1 and 4 ng/mL for glyphosate, glufosinate and AMPA, respectively, but the lowest fortification level for validation was set at 25 ng/mL. This fact does not comply with the SANTE document, which states that “The LOQ is the lowest spiked level of the validation meeting these method performance acceptability criteria” (identification, recovery and precision parameters). The other validation levels were 100, 500 and 2000 ng/mL in seven replicates each. Finally, the authors compared the results obtained with four different quantification methods (matrix/solvent calibration with or without ILIS). The calibration method using standard in solvent was applicable with an internal standard only because the recovery of AMPA without ILIS was lower than 50% for all spiking levels. All other recovery values were included in the range of 70–120% and an RSD ≤ 20% [25]. Good results were also obtained for egg samples. Experimental LOQs were 18, 6 and 30 ng/g, respectively. The fortification levels were 50, 100, 500 and 1000 ng/g, with the first one above LOQs in this case. Seven replicates were performed for each level, and calibration was conducted using a standard in solvent with ILIS. In this case, recovery values were included in the range of 70–120% and an RSD ≤ 20%.

The most complete method for analysis of polar pesticides was presented by Herrera et al. [3]. It includes 14 highly polar pesticides in several matrices of animal origin using a hydrophilic interaction liquid chromatograph by Obelisc-N (5 μm, 150 × 2.1 mm) (SIELC, Wheeling, IL, USA). Different quantitation approaches and clean-up procedures have been tested. The authors underlined that there were differences in terms of matrix effects between matrices of the diverse commodity groups but also among the same group (e.g., liver and kidney). For this reason, the use of isotopically labeled internal standards (ILIS) was strongly recommended. A mixture of H_2_O/MeOH acidified solvent was used for the extraction of all matrices. Liver, kidney, chicken meat and chicken eggs followed the same procedure that includes Oasis^®^ MCX cartridge (mixed-mode cation exchange) by Waters (Milford, New Zealand) for purification. The final dilution factor was 100. For cow milk, EDTA solution was added to chelate the metals possibly contained in the matrix to avoid interferences with the analytes. No purification steps were carried out. The final dilution factor was 50. For fat, hot water was added to the samples. Moreover, for this matrix, no purification steps were carried out, and the final dilution factor was 50. In some matrices, the MCX clean-up cartridge caused the partial loss of glufosinate and its complete loss in chicken eggs because of interactions between the sulfite functional group of the sorbent and the amino group in glufosinatestructure. For this reason, the authors validated eggs without this step. Linearity of the calibration curves, with an R2 > 0.98, was achieved for all pesticides. The deviation of the back-calculated concentration from the true concentration of each calibration standard, calculated to evaluate linearity according to Document SANTE [25], was always in the range of ±20%. The LOQs were generally fixed at 0.01 or 0.02 mg/kg, but for some compounds, they were higher. In some cases, they were intentionally validated at higher levels than other cases because of their lower sensitivity, especially in the case of qualifier ion transitions. Bromide could not be validated in the liver, kidney or chicken eggs because no blank samples were available. For all different matrices, the results of recoveries were included in the range 70–120% and RSDs were ≤20%. The only disadvantage of this method could be the high value of the dilution factor (50 or 100) with respect to the levels of the legal limits imposed by legislation.

## 3. Conclusions and Prospectives

The analysis of polar pesticides, especially in foods of animal origin, is not very common in control laboratories because they require specific single-residue analytical and instrumental analyses due to their chemical characteristics. Furthermore, a problem found in the literature concerns the lack of methods that include the simultaneous analysis of different polar pesticides. In fact, most of the published works only foresee the analysis of glyphosate and AMPA. Two methods only [3,30] are provided for the analysis of all analytes (glyphosate, glufosinate, MPP and N-acetyl glufosinate) required by the Commission Implementing Regulation (EU) 2021/601, as reported in Table 1. A factor that complicates the analysis of polar pesticides is the so-called “matrix effect”, defined as the influence of one or more interfering compounds co-extracted from the sample on the signal of the analyte that compromises its intensity or shape when samples are analyzed by mass spectrometry. This effect can lead to an increase or decrease in the chromatographic signal causing an overestimation or underestimation of the results. In almost all the works cited, the authors have conducted studies to evaluate this effect. Due to the variable results obtained, isotopically labeled internal standards (ILIS) or matrix matching calibration were required for quantification.

European laboratories require that validation of the analytical methods must follow the directives set out in the SANTE document [25]. The method must be tested to evaluate the linearity, average recovery, accuracy and LOQ. The summary of performance criteria data of the works in this review is reported in Table 2. To evaluate the linearity of the calibration curve, the deviation of the back-calculated concentrations with respect to the actual ones of each standard must be calculated. Despite this requirement, this approach has only been reported by Herrera et al. [3].

Different techniques for the analysis of polar pesticides have been described in this review.

A simple and fast method for the determination of glyphosate could be ELISA [8,9]. The disadvantage of this technique lies in the fact that, in the first paper, the extraction procedure needs 8 h for cooling and that the recovery study for lung and muscle matrices was completed at a concentration two times higher than the MRL. Moreover, the method has a repeatability value greater than 20%, as required by the SANTE regulation. Although Jhon et al. simplified the extraction procedure, they did not provide reliable data.

On the contrary, procedures using GC techniques produced good validation results for levels lower than MRL for glyphosate and AMPA [8,23]. Using GC, the disadvantage was related to the use of TFAA for derivatization because it reacts violently with water. Although the authors employed a mixture of TFAA and HFB, during the study, one analyst developed an allergic reaction to the derivatizing agent, and the authors underlined the use of all precautions to avoid contact with this molecule. Furthermore, in their study, Kruger et al. specify that icy TFAA should be added with caution.

The FI-MS/MS method [24] allows the analysis of glyphosate, as well as various other polar pesticides (Table 1). Furthermore, it greatly reduces the instrumental analysis time compared to other techniques. However, this can be considered a screening method since the EU guideline requires chromatographic separation for the identification of molecules.

Chiesa et al. have proposed the IC-HRMS method with a very simple extraction procedure, obtaining validation results compliant with the SANTE document. This method has many drawbacks, such as matrices not included in the EU program regulation (fish and muscle) and molecules with incomplete MRL definitions such as glufosinate. In fact, in Regulation (EC) 396/2005, glufosinate MRL is the sum of glufosinate isomers, its salts and its two metabolites [3[hydroxy(methyl)phosphinoyl]propionic acid (MPP) and N-acetyl-glufosinate (NAG)], expressed as glufosinate, but the authors omitted to include these two metabolites in the validation study. Finally, an advantage of using HRMS is the high specificity and the possibility of carrying out retrospective analyses.

The most common and widely utilized technique for the determination of polar pesticides in animal origin matrices is LC-MS/MS. Some of these methods involve a derivatization process. This step is often considered the main drawback of sample preparation due to its long process (for example, for Li et al., the derivatization phase time was set to one night) and unstable products, but it also has numerous advantages such as greater selectivity, sensitivity and possible identification and quantification of species in a single column, simultaneously [17,36]. Unlike direct analyses of polar pesticides, which require dedicated and innovative columns, the chromatographic separation of derivatized molecules can take place with columns in the C18 stationary phase. This can be considered a further advantage since the chromatographic stationary phases used for the underivatized analysis of polar pesticides often suffer from poor robustness performances, are quite expensive, delicate and require a time-consuming conditioning step [14,37]. All works reported show validation results compliant with the SANTE document for all analytes. This also applies to the work of Janson et al., but, similarly to Chiesa et al., did not include glufosinate metabolites in the method.

Different methods, without a derivatization step, are available in the literature, but only two of them are capable of extracting a wide range of polar pesticides besides glyphosate and AMPA. The first one is the QuPPe method [30], in which the extracts may contain high concentrations of co-extractive matrices that may contaminate the instruments [14]. In most cases, the different groups of analytes were validated at the respective MRL levels. In other cases, considering the complex definition of the MRL of the molecules like glufosinate included in the control plan for food of animal origin, the validated spiking levels were made at concentrations higher than MRL. The second method reported in the literature [3] allows for quantifying 14 highly polar pesticides with a simple and straightforward extraction procedure. The only disadvantage of this method could be the high value of the dilution factor (50 or 100 times) compared with the levels of legal limits imposed by legislation.

Glyphosate, AMPA and glufosinate in milk and egg matrices [32,33,34,35], are extracted with a simple procedure, and validation results satisfy the regulation parameters. The LOQs are below MRLs, but in the case of Zoller et al. and Narong et al., the LOQs reported are below the lower levels of validation. This fact does not comply with SANTE document requirements, which state that “The LOQ is the lowest spiked level of the validation meeting these method performance acceptability criteria” (identification, recovery and precision).

An advantage of the work of Narong et al. is the length of the acquisition methods, which was very short (6 min for milk and 12 min for egg, respectively).

In all LC-MS/MS methods reported, quantification occurs through the use of ILIS to compensate for the matrix effect, except for QUATs analysis [31], which involves matrix-matched calibration curves to overcome the problem. In conclusion, the area of polar pesticide analysis in food of animal origin still remains a rather neglected sector in the field of pesticide control in food commodities. The inherent difficulties in analyzing this small group of molecules and the need to use specific LC chromatographic columns make the diffusion of effective and reliable analytical methods somewhat limited with consequent scarce availability of data for risk assessment by competent authorities.

## Figures and Tables

**Table 1:** Main characteristics of methods used for the analysis of polar pesticides in food of animal origin.

Matrices	Analytes	ILIS	Extraction	Purification	Analytical Methodologies	Derivatization	Reference
Milk, beef, fish	Glyphosate	No	Centrifugation	-	ELISA	-	[20]
Liver, kidney, lung muscles	Glyphosate	No	Centrifugation	ultra-centrifugal filter	ELISA	-	[19]
		Yes		-	GC-MS/MS	TFE/TFAA	
Beef, muscle, kidney, cow milk, chicken eggs	Glyphosate AMPA	No	0.1 N HCl + chloroform, or 0.6% (*v*/*v*) acetic acid (milk)	cation exchange clean-up	GC-MS/MS	TFAA/HFB	[23]
Milk, kidney	10 analytes ^a^	Yes	Water	-	FI-MS/MS	-	[24]
Fish (bass), bovine muscle	Glyphosate, glufosinate, AMPA	Yes ^d^	methanol + acidified water	-	IC-HRMS	-	[16]
Cow′s milk	Glyphosate AMPA	Yes	Water/MeOH	-	LC-MS/MS	FMOC-Cl	[26]
Veal, chicken, pig liver	Glyphosate	Yes	Methanol/water (20/80 *v*/*v*).	SPE-WAX	LC-MS/MS	FMOC-Cl	[27]
Chicken, swine muscles	Glyphosate, AMPA	Yes ^d^	Water/DCM 2;1	CAX column	LC-MS/MS	FMOC-Cl	[28]
Liver, kidney bovine, milk	Glyphosate, AMPA, glufosinate	Yes	Water	-	LC- HRMS LC-MS/MS	Dansyl chloride	[29]
		Yes	Water/MeOH 50/50	-	LC-MS/MS	-	
Liver, kidney, muscle, milk, eggs, fat	24 analytes ^b^	Yes	Water/MeOH	ACN and C18 sorbent	LC-MS/MS	-	[30]
Chicken, pork, pork fat, beef, beef fat, egg, milk	Diquat, Paraquat, Chlormequat	No	ACN 0.5% formic acid + water 0.5% formic acid	HLB LP cartridge	LC-MS/MS	-	[31]
Milk	Glyphosate	Yes	water 0.1% formic acid	Methylene chloride	LC-MS/MS	-	[32]
Milk	Glyphosate, AMPA	Yes	water/methanol 1:1 (*v*/*v*) + 0.5 % formic acid	Oasis HLB cartridge	LC-MS/MS	-	[33]
Milk, eggs	Glyphosate, glufosinate, AMPA	Yes	50 mM acetic acid/10 mM Na_2_EDTA	Oasis HLB cartridge	LC-MS/MS	-	[34,35]
Liver, kidney, chicken meat and chicken eggs, milk and fat	14 analytes ^c^	Yes	H_2_O/MeOH acidificated	Oasis^®^ MCX cartridge (not for milk and fat)	LC-MS/MS	-	[3]

^a^ Ethephon, fosetyl-Al, glufosinate, glyphosate, AMPA, maleic hydrazide, chlormequat, diquat, mepiquat and paraquat. ^b^ AMPA, ethephon, fosetyl, glyphosate, glufosinate, HEPA, MPP, N-acetyl-AMPA, N-acetyl-glufosinate, N-acetyl-glyphosate, phosphonic acid, chlorate, perchlorate, aminocyclopyrachlor, amitrole chlormequat, chloridazon-desphenyl, cyromazine, mepiquat, morpholine, nereistoxin, trimethylsulfonium, propamocarb and melamine. ^c^ AMPA, ethephon, fosetyl, glufosinate, glyphosate HEPA, MPP, N-Acetyl-AMPA, N-acetyl-glufosinate, N-acetyl-glyphosate, phosphonic acid, chlorate, perclorate, bromide. ^d^ For glyphosate only.

**Table 2:** Summary of data of performance criteria. Validation, LOQs, recovery range and Cv%.

Reference	Validation Level Range (mg/kg)	LOQ Range (mg/kg)	Recovery Range	Cv%
[20]	No validation data presented			
[19]	100	-	91%	28%
[23]	0.01–1.0	0.01–0.1	70–120%	<20%
[24]	Validation data presented for plant origin matrices only			
[16]	0.01 and 0.05	0.004–0.006	70–120%	<20%
[26]	0.005	-	70–120%	<20%
[27]	0.025 and 0.250	0.025	70–120%	<20%
[28]	0.05–0.50	0.05	70–120%	<20%
[29]	10–250	From 0.010 to 0.25	70–120%	4–33%
[30]	0.005–0.2	-	70–120%	<20%
[31]	0.005–0.05	0.005	60–120%	<20%
[32]	0.01–2.5	0.01	70–120%	<20%
[33]	4	0.5 and 1	70–120%	<20%
[34,35]	0.025–2	0.001–0.03	70–120%	<20%
[3]	0.01–0.5	0.01–5	70–120%	<20%

## Data Availability

No new data were created or analyzed in this study. Data sharing is not applicable to this article.
